# The Role of 3D Printing in Regenerative Medicine: A Game-Changer in Tissue Engineering

**DOI:** 10.3390/ijms27062589

**Published:** 2026-03-12

**Authors:** Ameya Sharma, Vivek Puri, Kampanart Huanbutta, Tanikan Sangnim

**Affiliations:** 1 Chitkara University School of Pharmacy, Chitkara University, Himachal Pradesh, Baddi 174103, India; ameya.sharma@chitkarauniversity.edu.in (A.S.); vivek.puri@chitkarauniversity.edu.in (V.P.); 2 Department of Manufacturing Pharmacy, College of Pharmacy, Rangsit University, Pathum Thani 12000, Thailand; kampanart.h@rsu.ac.th; 3 Faculty of Pharmaceutical Sciences, Burapha University, Chonburi 20131, Thailand

**Keywords:** 3D printing, bioprinting, tissue engineering, biomaterials, scaffolds, hydrogel, wound healing

## Abstract

In regenerative medicine, three-dimensional (3D) printing provides precise spatial control over the fabrication of complex, biomimetic tissue constructs, enabling the production of architecturally defined and functionally tailored scaffolds. By enabling precise layer-by-layer deposition of cells, biomaterials, and bioactive compounds, 3D printing overcomes many limitations associated with conventional scaffold fabrication methods. This approach facilitates the development of tailored structures that mimic the mechanical, biological, and structural characteristics of native tissues, thereby enhancing cellular organization, proliferation, and differentiation. Extensive research in tissue engineering has led to the development of 3D-printed scaffolds for the regeneration of vascular, skin, bone, cartilage, and soft tissues. Advances in bioink formulations—including growth factor-loaded systems, decellularized extracellular matrix components, and natural and synthetic polymers—have further improved tissue-specific functionality. Moreover, multimaterial and multiscale printing strategies enable the fabrication of heterogeneous constructs with controlled porosity, mechanical gradients, and spatially regulated biological cues. Although vascularized tissue constructs remain a major challenge for clinical translation, recent bioprinting advancements have significantly accelerated progress in this area. Integration of computer-aided design with patient-specific imaging data has further strengthened the potential of 3D printing for personalized regenerative therapies. Despite these advances, challenges related to scalability, regulatory approval, and long-term functionality persist. Nevertheless, continued progress in printing technologies, biomaterials, and regulatory and standards frameworks is expected to drive the clinical adoption of 3D printing. Ultimately, 3D printing represents a transformative approach in tissue engineering, redefining strategies for functional tissue regeneration and translational regenerative medicine.

## 1. Introduction

Tissue engineering has experienced significant technological advancement with the emergence of three-dimensional (3D) printing, which enables the fabrication of complex, patient-specific scaffolds that closely resemble native tissue architecture [1]. The primary objective of tissue engineering is to develop biological substitutes capable of restoring or replacing damaged tissues. In this context, 3D printing provides precise spatial control over the deposition of biomaterials, extracellular matrix components, bioactive molecules, and living cells, thereby creating microenvironments that support cellular proliferation, differentiation, and tissue maturation [2,3].

One of the most critical challenges in tissue engineering is achieving functional vascularization. Conventional scaffold fabrication techniques often fail to reproduce the intricate vascular networks necessary for nutrient transport, oxygen diffusion, and long-term tissue survival after implantation [4]. Advanced 3D bioprinting techniques address this limitation by enabling controlled, layer-by-layer patterning of multiple cell types—such as endothelial cells, chondrocytes, and osteoblasts—within defined architectures. This capability supports the formation of vascularized, cartilage-like, and bone-like constructs that more closely mimic native tissue organization [5].

Beyond scaffold fabrication, additive manufacturing has become an important platform in healthcare. The layer-by-layer deposition process allows the production of geometrically complex structures with controlled porosity and internal lattice configurations that are difficult to achieve using traditional subtractive methods. These structural advantages enhance mechanical compatibility and biological integration in applications such as orthopedic implants, dental prostheses, and customized medical devices. In pharmaceutical sciences, 3D printing enables personalized dosage forms, including polypills and orally disintegrating systems, supporting patient-specific drug combinations and dose adjustments [6,7].

Recent advancements in multi-material and multi-cell bioprinting further expand the potential of tissue-mimetic constructs for regenerative medicine and organ replacement strategies [8,9]. In addition, 3D-printed anatomical models have improved surgical planning and training accuracy. Organ-on-a-chip platforms fabricated using 3D printing provide physiologically relevant models for drug screening, disease modeling, and toxicity assessment, offering improved predictive value compared to conventional two-dimensional cell cultures [10,11].

Despite these advances, several challenges remain before widespread clinical translation can be achieved. Printed tissues must exhibit mechanical properties comparable to native tissues, including appropriate elasticity, strength, and durability [12]. Long-term cell viability, integration with host immune and circulatory systems, and maintenance of functional performance after implantation are essential considerations. Scalability also remains a limitation, as producing large, fully functional tissues or whole organs requires precise cell distribution and sustained viability throughout the construct [13,14]. Furthermore, ethical and regulatory considerations must be addressed as bioprinting technologies approach clinical organ replacement applications [15].

Ongoing progress in biomaterials science, stem cell biology, and digital manufacturing technologies—including artificial intelligence-assisted design—continues to refine 3D printing strategies for tissue engineering. The integration of these approaches holds promise for overcoming current limitations and advancing toward clinically viable, patient-specific regenerative therapies [16,17].

Overall, tissue engineering represents an interdisciplinary field that integrates biology, materials science, and engineering to develop functional tissue substitutes. By addressing organ shortages and reducing reliance on donor transplantation, 3D printing-enabled tissue engineering offers a promising pathway toward personalized regenerative medicine [18,19].

### 1.1. Significance of 3D Printing in Tissue Engineering

Tissue engineering has been revolutionized by 3D printing, which allows for the exact development of biomimetic scaffolds with regulated mechanical characteristics, porosity, and architecture. Efficiently designing and depositing cells, growth factors, and bioactive materials in a patient-specific manner improves tissue regeneration and functional integration. Also, for uses in regenerative medicine and translational research, complex vascularized tissues may be produced with the help of modern bioprinting techniques [20]. The development of biocompatible scaffolds is an important area of research in tissue engineering. In order for cells to proliferate and organize into tissues with desired structures and functions, these scaffolds provide necessary support. Scaffolds that imitate the native extracellular matrix (ECM) have recently been developed, offering improved support for cell adhesion, proliferation, and differentiation [21].

### 1.2. Role of 3D Printing in Tissue Engineering

One of the most important aspects of tissue engineering is three-dimensional (3D) printing, which allows for the building of biomimetic scaffolds with the exact same mechanical and structural characteristics as natural extracellular matrices. It paves the way for the development of functional tissue design by enabling the exact spatial deposition of cells, biomaterials, and bioactive compounds using bioprinting methods. Regenerative medicine and translational therapeutic applications are further advanced by 3D printing’s ability to provide patient-specific design, quick prototyping, and the development of complex, vascularized structures. The advent of 3D printing has been a game-changer in the field of tissue engineering, allowing for the precise and accurate creation of intricate, personalized tissue constructions. Structural scaffolds that mimic the ECM of real biological tissues can be made with this method, which is also called additive manufacturing, by constructing tissues layer by layer using a computer model. Crucial for uses such as regenerative medicine, drug testing, and organ transplantation, these scaffolds enable cell proliferation, differentiation, and tissue development [22]. Transplant success rates are higher and rejection rates are lower thanks to the possibility of patient-specific tissues and organs made possible by 3D printing. To support bigger tissues and improve cell survival, it is necessary to be able to construct complex geometries, including vascular networks, that mirror the architecture of real tissues. Skin, bone, and cartilage are all examples of multi-layered structures that may be created using advanced 3D bioprinting processes that allow for the precise placement of different cell types [23]. Despite these benefits, there are still obstacles to overcome, such as the high costs and regulatory constraints related to the technology, and the need to ensure vascularization and cell survival within huge constructions. Ongoing research focuses on developing bioinks that better imitate tissues’ natural environment and integrating vascular networks. 3D printing has great potential as this area develops further to produce clinically useful tissues, lessen the need for organ donors, advance personalized medicine, and provide more precise models for drug testing and disease modeling, among other uses. In addition, 3D printing has the ability to supplant animal testing, which would be a better and more ethical way to conduct pharmaceutical research [24]. The technology has demonstrated outstanding success in creating simpler tissues and organ components, such as blood arteries, heart valves, and skin grafts, while completely functional printed organs are yet in the future. The capacity of 3D printing to precisely manufacture complex, functioning tissue structures has the potential to dramatically alter the landscape of tissue engineering and regenerative medicine, leading to better patient outcomes and the development of more tailored treatment options. Before widespread clinical use becomes a reality, critical hurdles must be overcome, such as scaling up, decreasing costs, and assuring long-term tissue viability [25].

## 2. Fundamentals of 3D Printing in Tissue Engineering

### Basic Principles of 3D Bioprinting

Tissue engineering 3D printing relies on digital model-based layer-by-layer production of biomimetic scaffolds, allowing for exact control of engineering, porosity, and mechanical characteristics to promote cell proliferation and tissue regeneration. To build functional tissue constructs using 3D bioprinting, one must first carefully deposit cell-laden bioinks, then undergo maturation and crosslinking to ensure structural stability and cellular viability. Three-dimensional bioprinting represents the merging of 3D printing techniques and tissue engineering. It is defined as the layer-by-layer precise positioning of living cells, biochemicals, and biological materials, with spatial control over the placement of functional components (like cells, extracellular matrix, and pre-organized microvessels) to fabricate three-dimensional structures. The core tool of this technology is the bioink, which is formed when living cells are integrated into a fluidic biomaterial [26]. This adaptation of 3D printing holds significant potential for regenerative medicine and reconstructive surgery by offering enhanced precision and potentially eliminating the need for donor site morbidity or immunosuppressive treatments [27].

Bioprinting is fundamentally based on the principles of tissue engineering, which is a branch of regenerative medicine. Tissue engineering applies the principles of life sciences and engineering toward developing biological substitutes that can restore, maintain, or improve tissue function or an entire organ. The primary aim of this discipline is to use a patient’s own cells to create an autologous graft. The success of tissue engineering relies on three foundational pillars: cells, scaffold, and signals (growth factors) [28].

The practical bioprinting process is divided into three main steps: pre-processing, processing, and post-processing. The pre-processing step uses computer-aided design and manufacturing (CAD-CAM) tools to define the desired structure. CAD-CAM is used to control both the overall shape (macroarchitecture) and the pattern of layer-by-layer deposition (microarchitecture) [29]. This design often utilizes medical imaging, such as computed tomography, involving image segmentation and mesh generation. The resulting design dictates how the bioink will be deposited. The bioink itself is composed of differentiated cells or stem cells mixed with biomaterials, which can include natural polymers like alginate, collagen, or gelatin, or synthetic polymers such as polycaprolactone. Specific growth factors, known as intercellular signals, may also be added during the preparation of the bioink [30].

The processing step involves classic 3D printers that have been adapted to receive cellular inks, utilizing methods like inkjet deposition, laser-assisted desorption, or microextrusion [31]. This phase demands exceptional control over printing parameters such as the speed of extrusion, viscosity of the ink, and the temperature of the extruder and the receiving plate to ensure the survival of the cells alongside the suitable rheology of the ink. Following printing, the final object undergoes post-processing, which includes a maturation step where it is kept under specific conditions inside an incubator [32]. This maturation requires the regular addition of growth factors and a daily culture medium supply; some authors refer to the time element of this stage as the fourth dimension, hence the term 4D bioprinting. The process’s success is ultimately judged by the cells’ survival and their ability to synthesize their extracellular matrix. A key advantage of bioprinting over classical tissue engineering techniques is the enhanced control of microarchitecture, allowing for a uniform distribution of cells and particles, which prevents accumulation at the bottom of the sample caused by gravity [33].

## 3. Materials for 3D Printing in Tissue Engineering

The field of 3D bioprinting is an adaptation of 3D printing techniques to tissue engineering, representing a key area within regenerative medicine. Regenerative medicine aims to develop biological substitutes that can restore, maintain, or improve tissue function or an entire organ, using a patient’s own cells to create an autologous graft [34]. Three-dimensional bioprinting specifically involves the layer-by-layer precise positioning of biological materials, biochemicals, and living cells, exercising spatial control over the placement of functional components (such as cells, extracellular matrix, and pre-organized microvessels) to fabricate three-dimensional structures. This technology holds significant potential to transform reconstructive surgery by enhancing precision and potentially eliminating the need for donor site morbidity or immunosuppressive treatments [35].

The materials used in 3D bioprinting are critical components, especially in tissue engineering and related applications like microbial engineering, and are generically referred to as bioinks. Bioink is formed when living cells (either differentiated cells or stem cells) are integrated into a fluidic biomaterial, which can be composed of synthetic or natural polymers [36]. Bioinks are hydrophilic gel materials, known as hydrogels, which possess a three-dimensional network structure that rapidly swells in water and retains a large volume of water without dissolving. Hydrogels are widely used in 3D bioprinting due to their high biocompatibility (often resulting in >80% cell viability) and adjustable physical and chemical properties [37]. Hydrogels are classified based on their material composition and source into two main categories, which are natural polymer hydrogels and synthetic polymer hydrogels.

### 3.1. Natural Polymer Hydrogels

Natural polymer hydrogels are central to 3D bioprinting in tissue engineering, serving as the primary bioink component that encapsulates living cells and provides a supportive scaffold; derived from hydrophilic substances of animal and plant origin, including extracellular matrix constituents, these materials form three-dimensional networks capable of rapid swelling and high water retention without dissolution, and their key attributes include biocompatibility, being generally non-toxic, non-immunogenic, and biodegradable; supporting cell viability commonly above 80%; and the ability to mimic in vivo microenvironments that foster cell or bacterial growth and proliferation, while enabling efficient bidirectional material exchange to supply nutrients and remove metabolic waste essential for sustaining embedded cells [38].

Natural polymer derivatives are generally classified into polysaccharide derivatives (e.g., hyaluronic acid, chitosan, alginate, agarose) and proteins and their derivatives (e.g., collagen, gelatin, silk fibroin, fibrin), as shown in Table 1 and Table 2. Natural polymer bioinks constitute a foundational category of biomaterials in extrusion-based 3D bioprinting, with sodium alginate, gelatin, collagen, hyaluronic acid, and chitosan representing five of the most prevalent examples [39].

#### 3.1.1. Critical Analysis of Specific Polymer Studies

Sodium Alginate: Valued for rapid ionic crosslinking with Ca^2+^, alginate provides excellent print fidelity. However, its lack of inherent cell-adhesive motifs often necessitates the incorporation of RGD peptides or blending with proteins. While studies show high cell viability (>95%), the resulting structures often suffer from long-term mechanical instability in physiological buffers due to the exchange of divalent cations [39].

Chitosan: Known for its antibacterial activity and hemostatic properties, chitosan is ideal for wound healing. Recent research successfully enhanced its performance by adding graphene oxide (GO), which improved cell adhesion and reduced degradation rates. Nevertheless, its slow gelation kinetics and requirement for acidic solvents can be detrimental to sensitive cell types if not carefully neutralized [40].

Gelatin and gelatin methacryloyl (GelMA): Gelatin’s thermo-responsive nature makes it an excellent sacrificial material, but its methacrylated derivative, GelMA, has emerged as a superior alternative. By providing photo-crosslinkable stability, GelMA resolves gelatin’s inherent structural weakness at body temperature while maintaining critical cell-binding motifs [39].

Collagen: As the most abundant protein in mammals, collagen provides an unparalleled biomimetic environment. However, the 30–60 min gelation time is a significant bottleneck for high-throughput 3D printing, often leading to poor shape fidelity unless reinforced with synthetic polymers or specialized support baths [41].

Silk Fibroin: Studies highlight its exceptional mechanical strength and low immunogenicity. Recent innovations include SF-based bilayer scaffolds that effectively reduce hypertrophic scarring by modulating wound contraction and inflammation [42].

Hyaluronic acid (HA): This polymer is a hydrophilic glycosaminoglycan. It contributes to cell proliferation, tissue hydration, and viscoelastic tuning but exhibits limited mechanical robustness and rapid degradation when used without modification. To address these challenges, HA is commonly incorporated as an additive to enhance viscosity and biocompatibility or chemically modified such as through methacrylation (HAMA) to permit light-mediated crosslinking and improve mechanical performance for extrusion printing [43].

#### 3.1.2. Challenges and Critical Deficits of Natural Polymer Bioinks

A primary obstacle to the clinical translation of natural polymer hydrogels is the mechanical–biological paradox, a conflict where the requirements for high-quality printing often oppose those for biological success. Achieving high shape fidelity and printability typically requires high material viscosity or dense cross-linking to ensure mechanical robustness and structural integrity. However, such rigid environments can impede essential cellular functions, including migration, proliferation, and differentiation, which flourish in softer, more biomimetic microenvironments. Consequently, single-component natural systems rarely provide an optimal balance, often resulting in scaffolds that either collapse during fabrication or lack the necessary biological cues for functional tissue regeneration [27].

Furthermore, the inherent nature of natural sources introduces significant batch-to-batch variability and structural limitations that complicate large-scale medical applications. Because these polymers are derived from plants or animals, factors such as molecular weight, purity, and composition can vary significantly between lots, leading to inconsistent rheological properties and unpredictable in vivo degradation rates [44]. For instance, materials like hyaluronic acid may degrade too rapidly under physiological conditions, potentially losing their role as a structural framework before the native tissue has sufficiently matured. This lack of stability also contributes to vascularization deficits; most natural hydrogels lack the mechanical strength to maintain the complex, open-lumen architectures required to form functional vascular networks, often leading to cell necrosis in larger, more dense tissue constructs [26].

### 3.2. Synthetic Polymer Hydrogels

Synthetic polymer hydrogels constitute a critical category of bioink materials in 3D bioprinting because they provide structural robustness, tunable physicochemical properties, and predictable behavior, attributes that often exceed those of natural hydrogels. As shown in Table 1, prepared from artificial polymers such as polyacrylic acid (PAA), polyvinyl alcohol (PVA), Pluronic F-127, and polyethylene glycol (PEG), these hydrogels offer longer service life, enhanced mechanical performance, and lower production costs [45]. Despite these advantages, they generally exhibit lower biocompatibility and limited biodegradability, requiring chemical modification or blending when applied in biological systems. Their controlled architecture and mechanical consistency make them widely used in environmental engineering, soft robotics, and increasingly in bioprinting [46].

Among these materials, PAA hydrogels have been extensively studied due to their high swelling capacity, pH responsiveness, and strong mechanical properties. They gel readily at mildly acidic pH but dissolve under highly acidic conditions, a feature useful for stimulus-responsive applications [47]. However, their carbon–carbon backbone and dense cross-linking hinder natural degradation, and unmodified PAA can display cytotoxicity. Therefore, functionalization through grafting with polysaccharides or proteins such as chitosan has been employed to improve mechanical performance, adsorption capacity, and biocompatibility. PVA hydrogels, by contrast, are known for their extensive hydrogen bonding, crystallinity, and resulting toughness. They retain high water content while offering good structural integrity, although cell adhesion and proliferation remain limited. Smart variants of PVA-based hydrogels, including supramolecular pH-responsive formulations, are being explored for future 4D bioprinting systems focused on environmentally responsive microbial release [48].

Other synthetic polymers, particularly Pluronic F-127 and PEG, are widely applied due to their favorable rheological behavior and compatibility with extrusion printing. Pluronic F-127 is a thermo-responsive polymer capable of reversible gelation within minutes, providing excellent printability, though with moderate cell viability and vulnerability to temperature-related stress [49]. Its performance improves significantly when blended with bioactive natural polymers such as collagen. PEG and its photopolymerizable derivatives, such as PEG dimethacrylate, are valued for high biocompatibility, rapid photogelation, high cell survival rates, and adjustable viscosity, supporting their use in biocatalytically active living materials. These polymers help overcome the mechanical limitations of many natural hydrogels [50].

To balance printability with biological performance, synthetic polymers are frequently incorporated into composite bioinks, including double-network (DN) hydrogels composed of two interpenetrating polymer networks. In DN systems, a stiff, densely cross-linked network dissipates energy under stress, while a second, flexible network maintains structural integrity [51]. Such combinations dramatically enhance mechanical strength, toughness, and recovery capacity compared to natural hydrogels alone. For example, incorporating PVA into a polyacrylamide/PVA DN hydrogel provides high tensile strength and resilience, enabling stable 3D-printed structures that resist collapse. In this way, synthetic polymers act analogously to structural steel in a building, imparting essential strength and durability, while natural polymers serve as the biologically functional interior environment that supports cellular activity [52].

**Table 1:** Comparison of main performance parameters of 3D bioprinting hydrogels (bioinks).

Hydrogel	Cross-Linking Method	Cross- Linking Time	Ink Density	Biological Survival	Key Advantages	Key Disadvantages	Ref.
**Natural Polymer Hydrogels**							
Chitosan	pH-mediated (CH_3_COOH)	2 h	3%	Inversely proportional to molecular weight	Strong adsorption capacity and low cost	Slow gel speed and poor mechanical properties; rarely used as sole material	[53]
Sodium alginate	Ionic (CaCl_2_)	10 min	1–2%	95%	Fast forming speed, low cost, and good stability	Poor cell adhesion; easy clogging at high concentrations	[54,55]
Hyaluronic acid (HA)	Photocrosslinking; pH-mediated	3 min	1.5%	–	Promotes proliferation and biofilm formation; fast gelation	Fast degradation and poor mechanical stability; structure limits application as hydrogel alone	[56,57]
Collagen	pH-mediated (NaHCO_3_)	1 min	0.3%	86%	Facilitates cell attachment; good printing ability	Poor mechanical stability; slow gel speed; easy to block	[58,59]
Gelatin	Temperature	5 min	5–20%	90% (after 45 days)	Thermosensitive crosslinking is reversible; promotes cell adhesion	Structurally unstable; unmodified gelatin has poor mechanical ability	[60,61]
Silk	–	–	80%	High biocompatibility and excellent mechanical stability	Low bacterial adhesion, which is not conducive to microbial reproduction	–	[62,63]
**Synthetic Polymer Hydrogels**							
Polyacrylic acid (PAA)	–	–	10–20%	Low cell viability	High water absorption; pH response	Refractory to degradation; high biological toxicity before modification	[64]
Polyvinyl alcohol (PVA)	–	–	30%	70%	Strong toughness and good biocompatibility	Low cell proliferation and adhesion	[65,66]
Pluronic F-127	Temperature	Minutes	0.8%	60%	Reversibility enhances printability	Damage from radiation and temperature to cells	[67,68]
Polyethylene Glycol (PEG)	Photo-polymerization	10 min	5–6%	90%	High biocompatibility and low cost	Soluble in organic solvents or water	[69,70]

**Table 2:** Comparative analysis of polymer suitability for 3D bioprinting.

Hydrogel	Degradation Rate	Effect on Vascularization	Optimal Field of Application
Chitosan	Moderate (about 21 days) (Enzymatic) [71]	Enhance vascularization and tissue regeneration thanks to their biocompatibility, biodegradability, and ECM-like structure [72]	Skin tissue engineering and wound healing [73]
Sodium Alginate	1 to 4 weeks depending on crosslinking density [74]	Low; requires bioactive additives [75]	Bone tissue engineering [76]
Hyaluronic Acid	A few days to over 42 days depending on crosslinking density, composition, and enzymatic activity [77]	High; promotes cell proliferation (Promotes endothelial lumen formation, branching, and vascular network development) [78]	Viscoelastic tuning; dermal fillers [79]
Collagen	High (Proteolytic), rapid degradation rate (often within days to a few weeks) [80]	Type-I collagen hydrogel promotes vascularization by preserving microvascular structure, stimulating sprouting, enabling capillary network formation, and upregulating pro-angiogenic genes [81]	Skin and wound healing applications, bone regeneration scaffolds, vascularized tissue constructs [82]
Gelatin/GelMA	Ranging from a few days to several weeks depending on crosslinking density, GelMA concentration, and enzymatic activity [83]	GelMA enables fabrication of porous, perfusable vascular networks that mimic the ECM and support endothelial cell growth and vessel formation [84]	Soft tissue engineering (skin, vascular) and, when enhanced with reinforcing agents, hard tissue regeneration (bone, cartilage) [85]
Silk Fibroin	Very Slow (Months) [86]	Enhance vascularization by providing a supportive matrix that promotes endothelial migration and vessel formation [87]	Silk fibroin is widely used in cartilage, bone, skin, and nerve regeneration due to its tunable strength, slow degradation, and ability to form structured 3D-printed scaffolds that support tissue repair [88]
Synthetic (PEG/PCL)	Highly tunable 3D-printable hydrogels allow controlled degradation from days to months by adjusting crosslinking and polymer composition [89]	3D-printed PEG/PCL hydrogels enhance vascularization by combining PCL’s structural support with PEG’s biocompatible environment, improving cell infiltration and capillary formation [90]	PCL and PEG-based hydrogels are used in bone, soft tissue, and neural engineering due to their strength, elasticity, and support for cell growth [91]

## 4. Fabrication of Scaffolds in Tissue Engineering

The fabrication of scaffolds is a foundational aspect of tissue engineering, providing the necessary architectural and biochemical support for cell is a foundational aspect of tissue engineering, providing the necessary architectural and biochemical support for cell growth and tissue regeneration. Three-dimensional (3D) printing techniques, particularly 3D bioprinting, enable the fabrication of complex tissue structures with high spatial accuracy and controlled architectural design [92].

### 4.1. Importance of Scaffolds in Tissue Regeneration

In order to promote cell adhesion, proliferation, and differentiation, scaffolds provide a three-dimensional structural framework that imitates the natural extracellular matrix. This framework is crucial for tissue regeneration. Degrading throughout time, they pave the way for the development of functionally restored tissue by supporting nutrition diffusion, waste disposal, and vascularization. Tissue engineering integrates principles from biology, materials science, and engineering to create functional biological substitutes capable of restoring or enhancing tissue function [93]. Within this framework, the scaffold serves as one of the three essential pillars alongside cells and biochemical signals because it provides the physical foundation upon which tissue formation occurs. Hydrogels and other polymeric materials frequently serve as scaffolds due to their ability to create hydrated three-dimensional networks that facilitate nutrient diffusion and waste removal, thereby supporting cell survival and proliferation. Their fluidic nature also enables encapsulation of cells to form bioinks suitable for advanced fabrication methods such as 3D bioprinting [94].

A key advantage of bioprinted scaffolds over those produced by traditional tissue-engineering methods is the enhanced ability to control spatial architecture. Precise deposition techniques allow uniform cell distribution and customized pore structures, preventing sedimentation or aggregation caused by gravity in conventional casting or seeding approaches [95]. This fine architectural control contributes to improved mechanical integrity, predictable mass transport, and optimized biological outcomes. Furthermore, scaffolds made from extracellular matrix–derived components or engineered synthetic materials can provide adhesion motifs and biochemical cues that regulate proliferation, differentiation, and tissue maturation [96].

In environmental engineering contexts, hydrogel-based scaffolds also serve protective and functional roles for embedded microorganisms. By providing a stable and hydrated microenvironment, hydrogels shield bacteria from toxic compounds, buffer harsh environmental fluctuations, and maintain metabolic activity even under high pollutant loads. This protective encapsulation enhances microbial resilience and enables their use in applications such as bioremediation, wastewater treatment, and pollutant degradation [97].

### 4.2. Methods for Scaffold Fabrication Using 3D Printing

**Figure 1:**
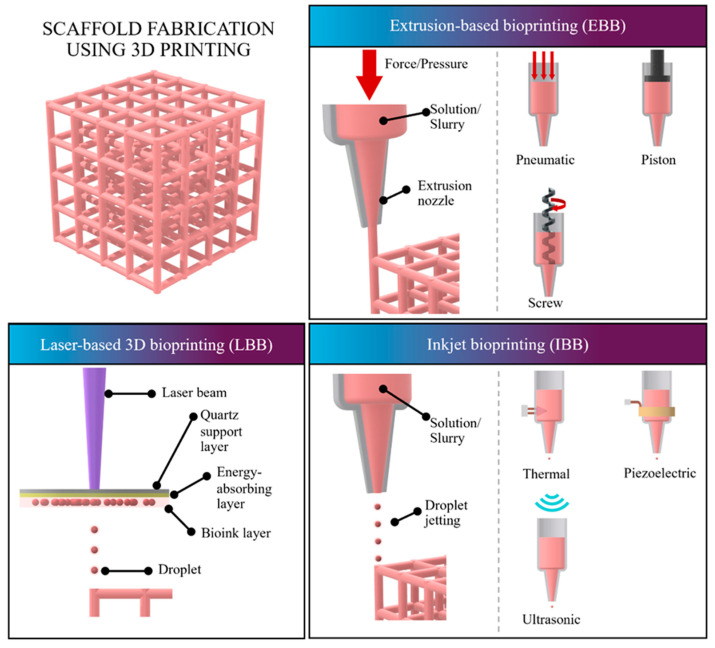
Schematic of 3D bioprinting techniques used in scaffold fabrication.

The key technologies utilized in 3D bioprinting are commonly classified into three main methods, often adapted from classic 3D printers to accommodate cellular inks: Laser-based 3D Bioprinting (LBB), Inkjet-based 3D Bioprinting (IBB), and Extrusion-based 3D Bioprinting (EBB), as shown in Figure 1 and Table 3. These technologies, previously reviewed and classified based on the microbial printing process, vary significantly in their mechanism, resolution, speed, and suitability for handling living cells and bioinks [98].

**Table 3:** Comparison of key 3D bioprinting technologies.

Technical Parameter	Laser-Based 3D Bioprinting (LBB)	Inkjet-Based 3D Bioprinting (IBB)	Extrusion-Based 3D Bioprinting (EBB)
Resolution	<10 μm	30–100 μm	>100 μm
Printing speed	Fast	Medium (Up to 10,000 drops/s)	Slow (10–50 μm/s)
Shape fidelity	High	Low	High
Supporting material required	Unnecessary	Necessary	Necessary
Material Viscosity Range	Medium (<300 mPa/s)	Low (3–12 mPa/s)	High (30 mPa/s to 6 × 10^7^ mPa/s)
Cell Concentration (Max)	High (up to 1 × 10^8^ cells/mL)	Low (<10^6^ cells/mL)	Very high
Cell Viability	95% (but low for microorganisms when UV is used)	>85%	40–80% (Negatively correlated with extrusion head diameter)
Gradient Printing Capability	No	Yes	Yes
Common Bioinks	Sodium alginate, collagen, gelatin, matrigel	Agar, alginate, fibrin, GelMA, polyethylene glycol, low viscosity living cell suspension	Alginate, hyaluronic acid, polyethylene glycol, agar, collagen, gelatin, pluronic, matrigel, fibrin, living cell suspension, composite materials
Key Disadvantage for Microorganisms	The nanosecond lasers often use ultraviolet (UV) light, which is fatal to bacteria by causing dimerization of thymine bases in their DNA, severely limiting application.	Causes severe thermal damage or mechanical damage, preventing widespread application.	Cell survival rate is lower due to the shear effect during extrusion, but impact on microorganisms is the mildest.
Cost	High	Low	Medium

#### 4.2.1. Extrusion-Based Bioprinting (EBB)

Extrusion-based bioprinting is currently the most promising and widely used bioprinting technology, primarily because of its wide compatibility with biomaterials and its unique gradient printing ability. In EBB, materials (like alginate or PEG) are extruded through a nozzle into a filament for layer-by-layer accumulation. Extrusion is driven by pneumatic, piston, or screw methods; pneumatic or piston methods are generally preferred for hydrogel bioprinting due to their low cost and strong universality, as screw extrusion’s strong shearing effect can compromise cell survival [99]. EBB is favored for its compatibility with a wide range of materials and its ability to achieve extremely high concentrations of printed cells. EBB also enables multi-channel printing, allowing for the precise control of multi-cellular and multi-material components simultaneously [100]. While its printing accuracy is lower than LBB (resolution >100 μm), and cell survival rates are typically 40–90% due to shear effects, the impact on microorganisms is the mildest of the three technologies, making EBB the most suitable method for microbial 3D bioprinting. EBB also benefits from high material viscosity compatibility (30 mPa/s to 6 × 10^7^ mPa/s) and versatile curing methods, including chemical crosslinking, light curing, and temperature-sensitive curing [101].

#### 4.2.2. Inkjet Bioprinting (IBB)

Inkjet-based bioprinting, also known as “drop-on-demand” printing, precisely controls the quantitative deposition of ink droplets in space to form the printed structure. Droplets are typically generated using thermal, piezoelectric, or ultrasonic droplet generators. IBB offers a high printing speed (approximately 1–10,000 drops/s) and a relatively high resolution (30–100 μm) [102]. Printers using IBB can often be transformed from commercial inkjet printers, lending them good operability and certain gradient printing capabilities. The primary constraint of IBB is its dependence on microfluidic principles, requiring bioinks with extremely low viscosity (3–12 mPa/s) and low cell concentrations (<10^6^ cells/mL). This low cell concentration limits its ability to support high-cell-density bio-embedding, and the resulting printed structures often suffer from poor continuity and low mechanical strength. Furthermore, IBB causes severe thermal or mechanical damage to microorganisms, preventing its widespread application in this area [103].

#### 4.2.3. Laser-Based Bioprinting (LBB)

Laser-based 3D bioprinting involves a system comprising a quartz support layer, an energy-absorbing layer, and a bioink layer. The process works by focusing a laser beam onto a small part of the quartz support layer, which generates a high-pressure bubble that pushes the bioink layer and forms droplets for deposition onto a substrate. LBB is a nozzle-free, non-contact process known for its advantages, including high resolution (<10 μm) and high shape fidelity. This technology can handle high cell densities (up to 1 × 10^8^ cells/mL) and hydrogel precursors with moderate viscosity (<300 mPa/s) [104]. Laser-guided direct writing, a novelty of this method, is the only technique currently available to directly place 10–1000 cells into arbitrary patterns with micrometer accuracy. However, a major disadvantage is that the nanosecond lasers used often employ ultraviolet (UV) light, which is fatal to bacteria by causing dimerization of thymine bases in their DNA, thus limiting LBB’s application in microbial bioprinting. LBB systems are also generally high in cost and require expensive photosensitive polymer materials [105].

## 5. Tissue Engineering in Wound Healing

The field of tissue engineering has the potential to revolutionize wound healing by creating viable alternatives to damaged skin through the use of concepts from engineering, materials science, and biology. Tissue engineering seeks to actively repair damaged or missing tissue, in contrast to conventional treatments that mainly target symptom management or short-term covering [106]. Scaffolds are the backbone of this method; they are three-dimensional constructs fabricated from biomaterials, either synthetic or natural, that lay the groundwork for the attachment, development, and differentiation of cells. In order to promote the body’s inherent healing mechanisms, these scaffolds are frequently mixed with stem cells, fibroblasts, keratinocytes, and other cell types, as well as bioactive substances like growth factors [107]. In addition to facilitating dermal and epidermal layer regeneration, the designed structures promote angiogenesis, the establishment of new blood vessels—an essential process for the long-term health of any given tissue. Modern innovations like smart hydrogels and 3D bioprinting make it possible to mimic the skin’s structure and reactivity to external stimuli with greater accuracy, which improves healing results even further [108]. Chronic wounds (such as diabetic ulcers), major burns, and other complicated skin ailments that do not react effectively to traditional treatments show particular promise for tissue-engineered skin replacements. Tissue engineering has the potential to revolutionize wound care and regenerative medicine by facilitating quicker healing, minimizing the probability of infection, and minimizing scarring [109].

### 5.1. Biopolymeric Scaffolds in Tissue Engineering

The biodegradability, biocompatibility, and ECM mimicking capabilities of biopolymeric scaffolds render them an essential component in tissue engineering. Hyaluronic acid, silk fibroin, collagen, gelatin, chitosan, alginate, and other natural polymers form the basis of these scaffolds, which facilitate migration, adhesion, proliferation, and differentiation. Because of their metabolic and structural characteristics, they foster an ideal setting for tissue regeneration [110,111]. The slow breakdown of biopolymeric scaffolds in the body enables the regenerated tissue to take over their function, eliminating the necessity for surgical removal. Nevertheless, they frequently necessitate cross-linking or blending with other polymers to improve their performance, as their mechanical strength and stability may be inferior to synthetic materials [112]. Electrospinning, freeze-drying, and 3D bioprinting are some of the fabrication techniques that can be used to create these scaffolds. This makes it possible to control the architecture, pore size, and porosity, all of which are important for nutrition diffusion and vascularization. Skin, bone, cartilage, and nerve tissue engineering hold great promise for biopolymeric scaffolds owing to their remarkable cell-interactive characteristics and low immunogenicity. Their continuous improvement is leading to solutions for tissue regeneration and repair that are increasingly effective, tailored, and biologically integrated [113,114].

#### 5.1.1. Chitosan

Chitosan, a deacetylated derivative of chitin obtained from crustacean exoskeletons, has been extensively investigated as a biomaterial for wound healing applications. Its reported properties include biocompatibility, biodegradability, low toxicity, and intrinsic antimicrobial activity [115].

Several in vitro studies have demonstrated that chitosan can promote fibroblast and keratinocyte migration, which are essential processes during tissue repair [116]. In addition, its cationic nature contributes to enhanced hemostasis through electrostatic interaction with negatively charged erythrocytes and platelets, facilitating clot formation [117]. Chitosan has also been reported to support cell adhesion and proliferation, although these effects are influenced by molecular weight, degree of deacetylation, and formulation parameters [118].

Mechanistically, chitosan may modulate the inflammatory phase by activating macrophages and stimulating the release of cytokines and growth factors; however, most evidence supporting this mechanism derives from in vitro and animal models, and translation to clinical settings remains limited [117,119].

From a formulation perspective, chitosan can be processed into hydrogels, films, sponges, and nanofibers, enabling adaptable scaffold architectures for different wound types [120,121]. Its porous structure provides a moist microenvironment that facilitates oxygen and nutrient diffusion. Furthermore, chitosan-based scaffolds are frequently combined with other biopolymers, growth factors, or bioactive agents to enhance regenerative performance.

Despite promising preclinical findings, robust clinical evidence for chronic wound management remains limited [122].

Recent studies have further explored functional modifications of chitosan-based scaffolds. In a study evaluating graphene oxide (GO) incorporation into chitosan–guar gum 3D scaffolds, the addition of GO improved cell adhesion, cytocompatibility, reduced degradation, and maintained high porosity [123]. While GO enhanced structural performance, concerns remain regarding long-term biocompatibility and potential oxidative stress.

Chitosan scaffolds augmented with polycaprolactone (PCL) nanofibers, fabricated via electrospinning combined with unidirectional freeze-drying, demonstrated improved cell growth, alignment, porosity, and water absorption [124]. A pH-responsive chitosan scaffold capable of loading and releasing exosomes promoted pro-proliferative and anti-inflammatory effects in a murine wound model [125]. Although this strategy exploits the dynamic wound microenvironment, reliance on exogenous exosomes introduces challenges related to storage stability, scalability, and regulatory approval.

Similarly, a biomacromolecule-based chitosan scaffold conjugated with glycyrrhizin-functionalized copper sulfide nanoparticles (GACuS) exhibited enhanced wound healing, moderate photothermal effects, and antibacterial activity under near-infrared irradiation [126]. In another study, incorporation of Quercus infectoria extract and silver nanoparticles (AgNPs) into chitosan scaffolds demonstrated improved wound-healing activity compared with silver sulfadiazine [127]. Despite enhanced antimicrobial efficacy, nanoparticle-related cytotoxicity and potential accumulation effects remain important considerations.

Additionally, a composite dressing containing amikacin sulfate and diopside nanoparticles embedded in chitosan exhibited strong antibacterial activity, fibroblast compatibility, and sustained drug release [128]. However, antibiotic-loaded systems must be evaluated carefully to mitigate the risk of antimicrobial resistance.

Lastly, *Discopodium penninervium* leaf extract-infused chitosan–PVA scaffolds showed improved tissue regeneration, accelerated wound closure, and enhanced antibacterial activity [129]. While plant-based functionalization may be advantageous in low-resource settings, variability in extract composition may affect reproducibility and standardization.

Collectively, these studies demonstrate the versatility of chitosan-based scaffolds in wound-healing applications. Nevertheless, most evidence remains preclinical, and further standardized in vivo and clinical investigations are necessary to establish long-term safety, reproducibility, and translational feasibility.

#### 5.1.2. Alginate

Alginate scaffolds may sustain a moist and cell-friendly environment, have moderate gelation qualities, and are highly biocompatible, making them ideal for use in tissue engineering. Alginate, a naturally occurring polysaccharide derived from brown seaweed, contains mannuronic (M) and guluronic (G) acid residues. When divalent cations, such as calcium, are present, they can create hydrogels [130,131]. The ionic crosslinking of alginate makes it an ideal material for the fabrication of injectable or moldable scaffolds that can accommodate many kinds of tissues. This is especially true for soft tissues like nerves, cartilage, and skin. Alginate scaffolds aid in wound healing by offering a moist environment that encourages the migration of cells and the regeneration of tissues. They also absorb excess wound exudate, which helps to avoid infection [132]. Chemical modifications (such as the addition of RGD peptides) or the incorporation of other biomaterials (such as collagen or gelatin) can improve cell attachment and proliferation in alginate, which does not naturally contain cell adhesion sites [133]. It is a flexible vehicle for regulated delivery in regenerative therapies due to its moderate gelation process, which is perfect for encasing cells, proteins, or medications. In general, alginate scaffolds are useful for in vitro tissue models and in vivo healing applications due to their bioactivity and flexibility, which promote cell viability and tissue development [134]. Using EXOs from human placenta-derived stem cells in an alginate hydrogel with a poly(ε-caprolactone) nanofibrous layer, one team of researchers investigates a hybrid nanofiber–hydrogel scaffold intended for regulated exosome (EXO) delivery. In a rat model, this scaffold enhanced collagen synthesis by 22% and 33% in comparison to controls, demonstrated regulated EXO release, enhanced cell proliferation, and faster wound healing [135]. Calcium alginate sulfate (Alg-S) was presented in a different study as an innovative essential for diabetic foot ulcer (DFU) wound treatment. Alg-S scaffolds showed improved mechanical, porosity, and swelling characteristics. These scaffolds encouraged normal skin tissue regeneration and collagen deposition, and they expedited wound healing in diabetic mice [136]. Concurrently, a study concentrated on a hydrogel scaffold made of polysaccharides, specifically alginate, pullulan, and hyaluronic acid, which offered the ideal milieu for mesenchymal stem cells (ASC) generated from adipose tissue. Good cell migration and adhesion were demonstrated in vitro, and ASC-seeded hydrogels greatly sped up wound closure in vivo, encouraging skin regeneration [137]. Zinc oxide nanoparticles (ZnO NPs) and Salvia abrotanoides essential oil (SAEO) were combined to create a novel electrospun nanofibrous wound dressing. This dressing improved tissue regeneration and collagen deposition, increased antimicrobial activity, and accelerated wound healing in full-thickness mouse wounds [138]. Furthermore, TEMPO-mediated oxidized sodium alginate (TOSA) was cross-linked with silk fibroin (SF) utilizing the EDC technique to create a scaffold. This scaffold demonstrated great porosity, good biocompatibility, and rapid disintegration, all of which aided in wound healing without secondary injury. Through improved cell activity, antibacterial qualities, and tissue regeneration, these various strategies show how advanced scaffolds might aid in wound healing [139].

Collectively, these studies demonstrate the versatility of alginate-based scaffolds in wound healing applications. Nevertheless, the use of alginate alone presents inherent limitations, particularly its poor intrinsic cell adhesion. As a result, many studies rely on blending alginate with other polymers or incorporating bioactive motifs to enhance biological performance. Another important consideration is the mechanical weakness of alginate and certain hybrid systems. Many alginate-based hydrogels remain relatively soft and may lack sufficient mechanical robustness, which could lead to structural instability or delamination under mechanical stress, especially in joint-related or high-mobility wound sites. Therefore, while alginate offers excellent biocompatibility and processability, further optimization of mechanical integrity and long-term stability remains essential for successful clinical translation.

#### 5.1.3. Gelatin

Tissue engineers frequently employ gelatin scaffolds because of their high biocompatibility, biodegradability, and resemblance to the ECM that comprises living organisms [140]. The inclusion of cell-binding motifs (such as the RGD sequence) that promote cell adhesion, proliferation, and differentiation is among collagen’s biological features that gelatin, a denatured version of the most prevalent protein in connective tissues, retains [141]. The use of freeze-drying, crosslinking, or 3D printing to transform gelatin into sponges, hydrogels, films, or electrospun fibers opens up a world of possibilities for scaffolding applications. Nevertheless, in order to enhance its structural stability and resistance to quick breakdown, gelatin is usually crosslinked chemically or physically (e.g., with glutaraldehyde, genipin, or UV light) due to its water solubility and mechanical weakness at body temperature [142]. Structural scaffolds made of gelatin are ideal for vascular, skin, bone, and cartilage tissue engineering because they promote cell proliferation and integration with the host tissue [143]. Their regeneration capabilities can be further enhanced by using them as vehicles for medicines, growth factors, or stem cells [144]. A number of cutting-edge nanofibrous scaffolds were developed to address severe wound healing concerns. Researchers developed multilayered nanofibrous scaffolds (MNSs) that were electrospun and encapsulated heparin sodium (HS) with high efficiency using a novel solvent system. This enabled sustained drug release and promoted diabetic wound healing by enhancing neovascularization and re-epithelialization [145]. In a different study, a PVA-gelatin-gentamicin scaffold that was covalently crosslinked and resembled the extracellular matrix had superior mechanical and antibacterial qualities, greatly speeding up in vivo healing [146]. A research project conducted by the team demonstrated improved wound closure and tissue regeneration in vitro and in vivo by incorporating bone marrow-derived mesenchymal stem cells (BMSCs) into electrospun PCL-gelatin nanofibers [147]. Furthermore, to increase mechanical strength, antibacterial activity, and biocompatibility, human amniotic membrane (hAM) was reinforced with gelatin and PPI-protected silver nanoparticles, providing a promising dressing for infected wounds [148]. A study combined bleomycin-loaded PCL/gelatin for targeted anticancer therapy with enrofloxacin-loaded PCL for antibacterial action to create a bilayer nanofiber scaffold for cutaneous squamous cell carcinoma (cSCC) postoperative care. Excellent tumor inhibition and wound healing were demonstrated by this multifunctional scaffold, confirming its clinical potential for complex wound care [149].

One of gelatin’s key advantages is its thermosensitive and reversible gelation behavior, which facilitates cell encapsulation and promotes cell adhesion features highly beneficial for wound healing, as they support early cellular infiltration and matrix remodeling. However, this thermoreversible property also contributes to structural instability at body temperature. Unmodified gelatin hydrogels typically exhibit poor mechanical strength and rapid degradation, which may lead to premature scaffold collapse in vivo. In wound healing applications, particularly in mechanically dynamic environments, this weakness can compromise structural support before sufficient tissue regeneration occurs. Therefore, although gelatin provides excellent biological functionality, mechanical reinforcement through crosslinking, methacrylation (e.g., GelMA), or hybridization with stronger polymers is often necessary to ensure durable and clinically reliable wound healing performance.

#### 5.1.4. Collagen

Collagen scaffolds are highly successful biomaterials for wound healing in tissue engineering. Their structural resemblance to the body’s natural ECM, great biocompatibility, and minimal immunogenicity make them a popular choice. In order to facilitate cellular adhesion, migration, proliferation, and differentiation, which are essential steps in wound healing, collagen, more specifically type I collagen, is a prime contender. Collagen is the principal structural protein in connective tissues, including skin [150]. Scaffolds made of collagen not only operate as mechanical supports, but they also direct tissue regeneration through interactions with cells and signaling molecules. Wound healing is accelerated and scar formation is reduced because they aid in maintaining a moist environment, facilitating angiogenesis (the development of new blood vessels), and modulating the inflammatory response [151]. To improve their mechanical strength and degradation rate, these scaffolds are frequently crosslinked or mixed with other materials, such as chitosan or hyaluronic acid. They can be made into films, membranes, hydrogels, or any number of different shapes. To enhance the healing process, collagen scaffolds can transport bioactive substances like growth factors (e.g., VEFG, PDGF) or antimicrobial chemicals. A staple of regenerative wound care treatments, bandages made of collagen are clinically used to treat burns, diabetic ulcers, and chronic wounds [152,153].

In order to improve wound healing, this study investigates a number of novel techniques. A biomimetic tri-layered artificial skin (TLAS) was created, which mimics the structure of real skin by using a porous collagen scaffold (dermis), a compact collagen membrane (bottom membrane), and an epidermis made of silica gel. In rat models, TLAS markedly accelerated re-epithelialization, collagen deposition, and skin appendage regeneration, demonstrating tremendous potential for treating deep skin injury [154]. The dual-crosslinking (EDC/NHS-DHT) technique also guaranteed high biocompatibility and bioactivity. In a different investigation, crucian carp skin was used to develop a biocompatible collagen-based hydrogel. The hydrogel was then evenly infused with luteolin. In vivo, this hydrogel promoted granulation tissue development, collagen deposition, and re-epithelialization by achieving a high drug loading efficiency (98%) and facilitating controlled luteolin release, which led to a 94% wound closure rate [155]. Furthermore, for diabetic wound healing, a hydrogel scaffold based on bioactive gelatin was created to encapsulate magnesium ascorbyl phosphate (MAP). By reducing oxidative stress and promoting collagen remodeling, MAP’s antioxidant qualities improved angiogenesis. Its promise as a successful treatment option for diabetic wound care was demonstrated by in vivo diabetic mouse models that showed faster wound closure (91.8% vs. 63% in controls) and elevation of important healing markers such as COL-1, CD31, VEGF, and CGRP [156].

Overall, collagen offers several important advantages, including excellent support for cell attachment, intrinsic bioactivity, and good printability for biofabrication applications. However, significant limitations persist. Native collagen hydrogels generally exhibit poor mechanical stability, slow gelation kinetics, and limited resistance to deformation under physiological stress, largely due to their low elastic modulus, which may result in structural collapse or contraction during in vivo remodeling. In extrusion-based bioprinting, collagen’s viscosity and fibrillogenesis behavior can also cause nozzle clogging and reduced shape fidelity. These mechanical weaknesses are particularly critical in wound healing applications involving high-mobility regions such as joints, where repeated mechanical stress may compromise scaffold integrity. Furthermore, rapid enzymatic degradation can diminish long-term structural support before adequate tissue regeneration occurs. Therefore, although collagen provides superior biological functionality, mechanical reinforcement through crosslinking, composite formulation, or hybridization with synthetic polymers is often necessary to achieve clinically durable and structurally robust wound healing outcomes.

#### 5.1.5. Cellulose

There has been recent interest in using cellulose, a naturally occurring polysaccharide mainly found in plant cell walls, in tissue engineering to promote wound healing because of its biocompatibility, great mechanical strength, and abundance [157]. Derivatives of native cellulose, such as bacterial cellulose, carboxymethyl cellulose (CMC), and hydroxypropyl methylcellulose (HPMC), have improved characteristics that make them useful in biomedicine. However, original cellulose itself is not bioactive and has no biological effect. Particularly beneficial for wound healing is bacterial cellulose, which is extremely pure, has a great capacity to retain water, and creates an environment that is both flexible and breathable while also being moist [158]. With these qualities, the wound site can be properly hydrated, epithelial cells can migrate more easily, and the likelihood of infection and scab development can be reduced. To further improve wound healing, cellulose-based scaffolds can be modified to include bioactive substances such as growth factors, antibiotics, or nanoparticles for controlled medication delivery [159]. Cellulose does not have any inherent sites for cell adhesion, although it is frequently altered or combined with proteins such as collagen or gelatin to enhance cell contact. Because of its structural tunability, it can be used to make nanofiber mats, membranes, hydrogels, or sponges that are designed to fit particular kinds of wounds or stages of healing [160,161]. When it comes to designing next-generation wound dressings and regenerative medicines, cellulose-based scaffolds are proving to be highly versatile, inexpensive, and environmentally friendly [162]. The development of several revolutionary nanofiber scaffolds for wound healing applications is investigated. Initially, carboxymethyl cellulose (CMC) combined with TiO_2_ nanoparticles and hydroxyapatite (HA) was used to create an antibacterial nanocomposite scaffold. With inhibition zones of 7.70 ± 0.02 mm against *S. aureus* and 5.20 ± 0.06 mm against *E. coli*, the scaffold showed strong antibacterial activity. With 100% wound closure as opposed to 88.40% in the control group, it demonstrated exceptional in vivo wound healing capability and maintained cell viability [163]. For diabetic wound healing, an inventive scaffold made of nanocellulose and filled with cerium oxide and silk fibroin nanoparticles was then developed. In comparison to commercial dressings, this scaffold had superior qualities like porosity, regulated degradability, and antibacterial activity, which reduced inflammation and encouraged collagen deposition, angiogenesis, and re-epithelialization in diabetic mice [164]. Another method involved the development of cellulose acetate (CA) electrospun nano-fibers encapsulating nano-chitosan and eucalyptus oil. These nanofibers exhibited significant antimicrobial activity, maintained safe levels of cell viability, and efficiently enhanced the in vivo wound-healing process by stimulating TGF-β and collagen production [165]. Lastly, propolis was used to develop electrospun nanofibers from microcrystalline cellulose (MC) and chitosan (CS), which demonstrated potent mechanical properties, hydrophilicity, biocompatibility, and antibacterial efficacy. Both acute and chronic wounds might benefit from the use of these functionalized nanofibers [166]. Considering them collectively, these studies demonstrate the variety and inventiveness of methods used to create bioactive nanomaterials for improved tissue regeneration and wound healing.

While the versatility and sustainability of cellulose-based scaffolds make them highly attractive for large-scale clinical use, the fundamental lack of inherent bioactivity in original cellulose remains a significant limitation that necessitates complex chemical modifications or protein blending. Furthermore, while the integration of inorganic nanoparticles provides impressive antibacterial and healing results in short-term animal models, the long-term metabolic clearance and potential systemic toxicity of these non-biodegradable components in human subjects remain under-researched. For these scaffolds to move from a prototype stage to a truly viable therapeutic platform, future studies must prioritize standardized safety evaluations of the functionalizing additives alongside the regenerative benefits of the cellulose framework.

#### 5.1.6. Silk Fibroin

A natural protein extracted from the silkworm Bombyx mori, silk fibroin, is an outstanding biomaterial for wound healing in tissue engineering. It possesses a unique blend of qualities, including biocompatibility, mechanical strength, biodegradability, and minimal immunogenicity [167]. Its structure is composed of crystalline parts that provide strength and amorphous sections that provide elasticity. This makes it very adaptable and may be used in many different forms of scaffolds, such as films, sponges, hydrogels, and electrospun fibers. Important steps in skin regeneration, including cell adhesion, proliferation, and migration, can take place on silk fibroin scaffolds, which are used in wound healing applications. Silk fibroin aids in the rapid stabilization of wounds and the promotion of new tissue creation due to its intrinsic hemostatic characteristics and its support of angiogenesis [168,169]. It works in tandem with the body’s natural process for repairing damaged tissues because, unlike other synthetic materials, it breaks down into harmless amino acids over time. To speed up the healing process and avoid infections, its surface can be simply changed to improve cellular connections or loaded with therapeutic substances such as growth hormones, antibiotics, or anti-inflammatory drugs [170]. In addition, silk fibroin is ideal for use as a wound dressing because of its pliability and transparency, which permit continuous monitoring of the wound without removing the dressing. In conclusion, silk fibroin is an extremely encouraging natural substance that aids in wound healing and tissue regeneration via structural and molecular mechanisms [171]. Deep tissue damage and bacterially infected skin wounds continue to be significant challenges in wound care and regenerative medicine. An array of advanced scaffolds and dressings based on silk fibroin (SF) have been created to address these challenges in order to minimize scarring, encourage healing, and stop infection [172].

Using a nanofibrous scaffold that incorporates Lactobacillus reuteri and tannic acid–Fe(III) chelates (Mbr/L@FeTA), one novel method achieves over 99% bactericidal efficacy against *S. aureus* and *E. coli* while providing strong antibacterial characteristics without the use of antibiotics. This scaffold exhibits outstanding mechanical performance and biocompatibility while also promoting cell development and modulating inflammation [173].

A bioactive hydrogel made of SF and GelMA was created with curcumin (Cur) for long-lasting antibacterial activity in order to promote tissue regeneration and manage infection. With its porous structure, enhanced mechanical strength, and support for cell adhesion and proliferation, this GelMA-SF@Cur hydrogel released around 55% of curcumin over the course of 24 h while successfully preventing bacterial growth [174].

A bilayer scaffold comprising an SF/hyaluronic acid (HA) dermal layer and a silk fibroin nanofiber (SNF) epidermis was designed to address hypertrophic scarring from deep wounds. Through the modulation of wound contraction, inflammation, and collagen deposition, this bilayer design reduced scar formation while promoting vascularization and tissue regeneration [175]. A green technique was used to create a photothermal-responsive membrane (PVA-SF/CuS) that incorporates SF-copper sulfide nanoparticles for infected wound settings. Under near-infrared irradiation, this nanofiber membrane demonstrated potent antibacterial activity, decreased inflammation, encouraged angiogenesis, and sped up healing [176]. A three-layer composite dressing with asymmetric wettability was developed to control wound exudate and avoid scarring. It consists of an inner layer that is hydrophobic and loaded with retinoic acid, a transitional layer made of silk fibroin, and an outside layer made of hydrophilic cotton. In burn and hypertrophic scar models, this structure improved fluid flow and cell migration, which sped up wound healing and decreased scarring [177].

Last but not least, a highly porous (~92.5%) bioactive SF scaffold including HA and SNFs was created to replicate extracellular matrix architecture, motivated by the fetal skin’s ability to repair without leaving scars. Within four weeks, the scaffold achieved 98.2% wound healing while avoiding the formation of scars by promoting endothelial cell proliferation and directing collagen organization in vivo [178].

The transition of silk fibroin from a traditional textile material to a high-tech regenerative scaffold represents one of the most successful examples of natural polymer engineering. The reported ability to achieve near-total wound closure and scar inhibition by mimicking fetal skin architecture is particularly groundbreaking. However, a critical bottleneck remains in the lack of standardization regarding the degumming process of raw silk; residual sericin can trigger inflammatory responses, yet over-processing often compromises the very mechanical strength that makes SF desirable. Furthermore, while the antibiotic-free bactericidal scaffolds and photothermal-responsive membranes show exceptional promise for treating multi-drug resistant infections, their clinical translation will likely face rigorous regulatory scrutiny regarding the consistency of “living” components like *Lactobacillus reuteri* or the systemic safety of copper-based nanoparticles. We believe future research must focus on optimizing these green extraction methods and establishing standardized degumming protocols to ensure safety without sacrificing structural performance.

### 5.2. Bioprinting in Tissue Engineering

With the use of bioprinting, a state-of-the-art tissue engineering technique, three-dimensional structures packed with cells can be precisely created by layer-by-layer deposition of bioinks, which are made of living cells, biomaterials, and bioactive substances. Complex tissue concepts that closely resemble natural biological tissues in both shape and function can be developed employing this technology [179]. Depending on the required resolution, material viscosity, and application, different bioprinting techniques such as inkjet, extrusion, laser-assisted, and stereolithography offer varying benefits. Natural polymers that enhance cell viability and encourage tissue-specific functions, such as collagen, silk fibroin, gelatin, or alginate, are frequently included in bioinks. In a variety of tissues, such as skin, bone, cartilage, cardiovascular, and neurological tissues, bioprinting has demonstrated encouraging uses, providing answers for wound healing, regenerative medicine, and disease modelling [180,181]. Customizing scaffolds in terms of geometry, porosity, and mechanical characteristics is one of its primary benefits, as it makes it possible to develop organ models and implants that are unique to each patient. Achieving functional vascularization, providing long-term mechanical stability, and overcoming regulatory obstacles for clinical application are still challenges to be overcome despite enormous advancements [182]. Future advancements like 4D bioprinting, multi-material systems, and AI integration, however, offer the potential to improve the clinical utility and functionality of bioprinted tissues, establishing bioprinting as a game-changing strategy for organ regeneration and customized medicine [183]. This series of research investigates cutting-edge methods for tissue engineering and wound healing using 3D bioprinting and the creation of bioinks [184]. Bifunctional hydrogel inks have been developed in one study to stimulate the formation of M2 macrophages and exosomes (M2-Exo), which improve wound healing by triggering the PPAR and JAK/STAT pathways. The collagen/decellularized extracellular matrix (COL@d-ECM) bioink in which these exosomes were encapsulated supported skin cell survival and promoted epidermal remodeling, which in turn accelerated wound healing and hair follicle regeneration in vivo [185].

Another work explored the challenge of using phosphosilicate calcium bioglasses (PSCs) in bioinks to enable nutrition exchange and replicate the mechanical characteristics of native skin. By producing full-thickness skin replacements that encouraged angiogenesis and improved vascularization in a rat wound model, this method greatly increased mechanical strength and biocompatibility and provided a potential method for vascularized skin transplants [186]. An optimized alginate–gelatin–DEAE cellulose bioink, with and without fibrinogen, was tested in a study that examined how the scarcity of donor skin has prompted the development of skin substitutes through 3D bioprinting. In comparison to acellular and dermal-only structures, the fibrinogen-containing formulation demonstrated its promise for regenerative medicine by reducing inflammation, promoting faster wound healing, increasing collagen deposition, and enhancing angiogenesis [187].

To combat the prevalent issues of infection, oxidative stress, and inflammation in the process of wound healing, a bilayered multifunctional scaffold called GQL/dGQue was created. This scaffold, which was created using 3D bioprinting, has potent antibacterial, free radical-scavenging, and anti-inflammatory qualities and resembles the structure of normal skin. It showed great promise for therapeutic skin restoration in vivo by dramatically speeding up wound healing, preventing infection, and encouraging vascularization and hair follicle regeneration [188]. Using in situ 3D bioprinting, a MoS_2_-accelerated gelling hydrogel scaffold was developed to improve wound healing in chronic diabetic wounds. Rapid hydrogel formation was rendered possible by the MoS_2_ nanosheets, which also provided photothermal and antioxidant qualities that reduced oxidative stress, encouraged wound closure, and got rid of bacterial infections, making this a promising treatment option for chronic diabetic wounds [189]. Platelet-rich plasma (PRP) was also incorporated into alginate–gelatin composite hydrogel bioinks using in situ 3D bioprinting. Compared to conventional bioinks, the inclusion of PRP enhanced cellular activity, encouraged angiogenesis, and reduced inflammation, hastening wound closure. Rapid, customized wound healing was supported by the regenerative capacity of this PRP-enhanced bioink [190].

The development of a composite scaffold containing salvianolic acid B (SAB) was a unique strategy for diabetic wound healing. With its potent anti-inflammatory, proangiogenic, and antioxidant qualities, this 3D bioprinted scaffold promoted collagen deposition, granulation tissue regeneration, and wound healing, providing an intuitive yet effective treatment for diabetic wounds [191]. For skin tissue engineering, a biomimetic composite bioink was developed by combining chitosan nanoparticles (CSNPs) and curcumin-loaded nanoparticles (Cur-NPs) with GelMA. This composite scaffold demonstrated potent antibacterial qualities, regulated drug release, and adjustable physical attributes. It demonstrated its potential for efficient wound healing in vivo by promoting cell proliferation and lowering microbial infections [192]. Another study developed a wound scaffold that combined niosomes (Nio), chitosan-alginate hydrogel, and the antibacterial plant component Barijeh (Bar). The clinical promise of the Nio-Bar@CS-AL scaffold for treating infections was suggested by its strong antibacterial activities, which greatly reduced bacterial growth and biofilm formation while facilitating wound healing, collagen deposition, and inflammation reduction in vivo [14]. Finally, self-assembled F127 diacrylate (F127DA) micelles were added to a dynamic hyaluronate hydrogel to enhance its viscoelasticity and gelling kinetics. This hydrogel promoted fibroblast proliferation and keratinocyte production by enabling accurate 3D bioprinting of cell-rich structures. By encouraging angiogenesis, extracellular matrix synthesis, and inflammation suppression, it sped up healing in a full-thickness mouse skin wound model, showing potential for tissue regeneration [193,194].

## 6. Challenges in 3D Printing for Tissue Engineering

3D printing has become a promising technology in tissue engineering; nonetheless, numerous challenges persist that hinder its effective transference from laboratory research to clinical practice. One of the main challenges involves developing appropriate bioinks that can concurrently offer excellent printability, biocompatibility, biodegradability, and sufficient mechanical strength, as materials optimized for printing often lack biological functionality, whereas biologically active materials frequently exhibit inadequate shape fidelity. During the bioprinting process, cells experience mechanical shear stress, thermal fluctuations, and chemical crosslinking, which may negatively impact cell viability, proliferation, and sustained functionality [194]. Furthermore, the majority of bioprinted constructs demonstrate inadequate mechanical robustness and structural integrity in comparison to native tissues, thereby significantly constraining their utilization in load-bearing tissues such as bone and cartilage. An additional significant challenge is the absence of functional vascular networks within 3D-printed constructs, which impedes effective diffusion of oxygen and nutrients and results in cell necrosis in dense or intricate tissues. Recreating the native tissue microenvironment with high spatial precision, multiple cell types, and intricate hierarchical structures continues to pose significant technical challenges. Furthermore, challenges concerning reproducibility, scalability, long-term integration with host tissues, immune responses, and the lack of standardized regulatory guidelines present substantial obstacles to the broad clinical implementation of 3D-printed tissue-engineered constructs [195,196].

## 7. Recent Advances and Innovations

Recent advancements in 3D printing, especially 3D bioprinting, have significantly revolutionized regenerative medicine and tissue engineering by enabling the development of intricate, biomimetic tissues and organ constructions that closely mimic natural architecture and functionality [197,198]. The advancement of next-generation bioinks, comprising exosome-enriched formulations and decellularized extracellular matrix (dECM)-based systems, has markedly improved cell survival, immunomodulation, and tissue-specific differentiation. Porous β-tricalcium phosphate (β-TCP) scaffolds combined with macrophage-derived exosomes have exhibited prolonged bioactive release, promoting osteogenesis and angiogenesis via regulated immunomodulation [199,200,201]. Conversely, dECM bioinks produced through perfusion or immersion decellularization depend chiefly on collagen crosslinking for structural stabilization and are especially appropriate for extrusion-based and digital light processing methods, providing tissue-specific biochemical signals and advantageous rheological characteristics [202,203].

Recent technological advancements, such as two-photon polymerization (TPP) and laser-assisted droplet-based bioprinting, have made micro- and nanoscale accuracy possible, allowing for the development of thick, vascularized constructions that can serve as models for the kidney, liver, and lung [202]. At the same time, process optimization, defect detection, and scaffold architecture design have been transformed by integrating Artificial Intelligence (AI) and Machine Learning (ML) into computer-aided design and closed-loop control systems. Particle swarm optimization, generative adversarial networks, and other state-of-the-art algorithms allow for reduced experimental iteration while optimizing mechanical strength and porosity multi-objectively [204,205]. 4D bioprinting goes beyond static constructions by introducing stimuli-responsive materials that can change their structure dynamically or release biofactors under regulated conditions. The development of bioprinted organoids, tissues, and patient-specific treatment platforms is being sped up by these interdisciplinary innovations [206,207].

## 8. Conclusions and Future Trends

3D printing has swiftly become a groundbreaking technology in tissue engineering, enabling the production of intricate, patient-specific tissue constructs with exceptional accuracy and consistency. Through the integration of digital design tools, sophisticated bioprinting methods, and an extensive selection of biomaterials, 3D printing enables the fabrication of scaffolds and tissue models that accurately replicate the architecture, mechanical characteristics, and biological microenvironment of native tissues. Its versatility spans a wide range of applications, including bone and cartilage regeneration, skin and soft tissue engineering, vascularization, organ fabrication, and in vitro disease modeling for drug testing. The incorporation of living cells, growth factors, and bioactive molecules into bioinks further augments the functional and regenerative potential of printed constructs, advancing toward the objective of fully functional tissue replacements. Despite these encouraging developments, numerous obstacles continue to hinder the complete clinical implementation of 3D-printed tissues. These encompass the creation of vascularized and innervated constructs capable of sustained long-term viability, precise regulation of mechanical and degradation characteristics, preservation of cell viability and functionality throughout and following the printing process, and effective management of intricate regulatory pathways for clinical application. Material selection, printing resolution, and scalability continue to be essential considerations in the design of constructs appropriate for clinical applications. Tackling these challenges necessitates continuous interdisciplinary collaboration among materials scientists, bioengineers, clinicians, and regulatory authorities.

Recent advancements in bioprinting techniques, including multi-material and hybrid printing, intelligent bioinks, and stimuli-responsive scaffolds, are progressively addressing these limitations. Furthermore, the integration of 3D printing with emerging technologies such as stem cell biology, computational modeling, artificial intelligence, and targeted delivery of tissue-specific growth factors is poised to expedite the advancement of personalized, functional tissues and organs. In the future, 3D printing is expected to assume a pivotal position in regenerative medicine, facilitating personalized therapies, diminishing dependence on donor tissues, and enhancing the overall efficacy and success rates of tissue repair and organ transplantation. In conclusion, sustained progress within this domain is poised to revolutionize the field of tissue engineering, thereby facilitating the transition from preclinical investigations to clinical implementation and presenting novel therapeutic avenues for patients necessitating intricate tissue reconstruction.

Transforming the future of tissue engineering, three-dimensional (3D) printing allows for precise spatial control over cells, biomaterials, and bioactive cues. One of the long-standing challenges in tissue fabrication has been the difficulty in accurately reproducing the structural, mechanical, and biological complexity of natural tissues. However, this should change as additive manufacturing technologies advance and the fields of bioprinting, biomaterials science, and regenerative biology come together. Progress in the field will be directed towards developing bioinks of the future that can adapt to the biological microenvironment in real-time, in addition to being printable and mechanically strong. It is expected that post-printing cell survival, differentiation, and tissue maturation will be improved by smart bioinks that include stimuli-responsive polymers, growth factor gradients, and immunomodulatory components. Integrating bioinks derived from dECM can also enhance biomimicry and functionality tailored to individual tissues. Making artificial tissues that are both vascularized and innervated is another important step forward. A hierarchical vascular network is crucial for nutrition transfer and long-term tissue survival. New technologies that combine 3D printing with microfluidics, sacrificial templating, and multi-material printing might make this possible. Concurrently, developments in high-resolution printing methods, such as volumetric bioprinting and two-photon polymerization, will make it easier to fabricate intricate microarchitectures on a cellular and subcellular level. In terms of translation, 3D printing holds significant potential for the development of customized, on-demand tissue prostheses in the field of tissue engineering. Customized scaffolds and tissues will be possible to meet the unique anatomical and clinical needs of each patient by combining patient-specific imaging data with AI-driven design optimization. For clinical acceptance to occur, automation, standardized regulations, and scalable production methods are also essential. Regenerative medicine aims to experience a radical transformation as 3D printing moves from a prototype tool to a therapeutically viable manufacturing platform. This will pave the way for functional tissue replacement, disease modeling, and precision therapies, among other potential game-changers.

## Data Availability

No new data were created or analyzed in this study. Data sharing is not applicable to this article.
